# Supplementary material for: Cardioprotective Effects of Ursodeoxycholic Acid in Isoprenaline-Induced Myocardial Injury in Rats
Source: Biomolecules. 2024 Sep 26;14(10):1214. doi: 10.3390/biom14101214 (PMC11506574; doi:10.3390/biom14101214)
Supplement: Supplementary file 1 [file biomolecules-14-01214-s001.zip › biomolecules-3164810-supplementary.pdf]

Table S1. Primer sequences used for qRT-PCR amplification.

Gene	Sequence	Length
NFκB	F AGTGCGGGACCCATCAGGCA<br>R GCAGTGTTGGGGGCACGGTT	84
TNFα	F AGCCCTGGTATGAGCCCATGTA<br>R CCGGACTCCGTGATGTCTAAGT	109
BAX	F CCGCGTGGTTGCCCTCTTCT<br>R AGGCCTTCCCAGCCACCCTG	159
